# P47 - Does vitamin D status in childhood modify risk for asthma development by altering susceptibility to severe respiratory infection and allergic sensitisation?

**DOI:** 10.1186/2045-7022-4-S1-P102

**Published:** 2014-02-28

**Authors:** Elysia Hollams, Guicheng Zhang, Barbara Holt, Merci Kusel, Peter Sly, Patrick Holt, Prue Hart

**Affiliations:** 1 Telethon Institute for Child Health Research, University of Western Australia, Perth, Australia; 2 University of Western Australia, Perth, Australia; 3 Queensland Children's Medical Research Institute, Brisbane, Australia

## Background

Vitamin D has entered the spotlight in the search for preventive treatments against asthma and allergic disease due to its immune-modulating functions, shown in experimental models to include promotion of immune tolerance and boosting protection against infection. Vitamin D inadequacy is common, but disparate findings from cohort studies have had a polarising effect on the scientific community regarding the wisdom of advocating vitamin D supplementation for protection against asthma and allergic disorders. We have previously found that in the high-risk Western Australian CAS cohort (selected due to positive parental atopic history), the combination of multiple severe lower respiratory infections and sensitisation to inhaled allergens by age 2 profoundly increased risk of asthma development by age 5.

## Aims

To determine whether vitamin D levels between birth and age 10 years in the CAS cohort are related to frequency of severe respiratory infections in early childhood, allergic sensitisation, and development of asthma by age 5 or 10 years.

## Methods

We used UPLC/MS/MS (accuracy confirmed with DEQAS standards) to measure 25(OH)-vitamin D3, 3-epi-25(OH)-vitamin D3 and 25(OH)-vitamin D2 from cryobanked plasma samples collected from CAS participants at birth, then at 6 months and 1, 2, 3, 4, 5 and 10 years. CAS participants were visited by the study physician up to age 5 years for every episode of respiratory infection.

## Results

Vitamin D3 inadequacy was common amongst cohort participants, and as expected was highest amongst participants from whom blood was collected in winter; deseasonalised vitamin D3 was calculated for longitudinal comparisons. In all assessments between 6 months (n=233) and 4 years of age (n=189) the majority of participants had inadequate vitamin D3; 51%-67% of participants had insufficient vitamin D3 (50-75 nmol/L) while 18%-28% were vitamin D3 deficient (<50 nmol/L). Analyses addressing the aims are underway and will be presented at the Meeting.

